# Testicular histopathology, semen analysis and FSH, predictive value of sperm retrieval: supportive counseling in case of reoperation after testicular sperm extraction (TESE)

**DOI:** 10.1186/s12894-018-0379-7

**Published:** 2018-07-04

**Authors:** Lucio Gnessi, Filomena Scarselli, Maria Giulia Minasi, Stefania Mariani, Carla Lubrano, Sabrina Basciani, Pier Francesco Greco, Mikiko Watanabe, Giorgio Franco, Alessio Farcomeni, Ermanno Greco

**Affiliations:** 1 Department of Experimental Medicine, Section of Medical Pathophysiology, Food Science and Endocrinology, Sapienza University of Rome, Policlinico Umberto I, 00161 Rome, Italy; 2 Centre for Reproductive Medicine, European Hospital, Rome, Italy; 3 Department Gynaecological-Obstetrical and Urological Sciences, Sapienza University of Rome, Policlinico Umberto I, 00161 Rome, Italy; 4 Department of Public Health and Infectious Diseases, “Sapienza” University of Rome, Rome, Italy

**Keywords:** Testicular sperm extraction (TESE), Testicular biopsy, FSH, Semen, Sperm retrieval

## Abstract

**Background:**

To provide indicators for the likelihood of sperm retrieval in patients undergoing testicular sperm extraction is a major issue in the management of male infertility by TESE. The aim of our study was to determine the impact of different parameters, including testicular histopathology, on sperm retrieval in case of reoperation in patients undergoing testicular sperm extraction.

**Methods:**

We retrospectively analyzed 486 patients who underwent sperm extraction for intracytoplasmic sperm injection and testicular biopsy. Histology was classified into: normal spermatogenesis; hypospermatogenesis (reduction in the number of normal spermatogenetic cells); maturation arrest (absence of the later stages of spermatogenesis); and Sertoli cell only (absence of germ cells). Semen analysis and serum FSH, LH and testosterone were measured.

**Results:**

Four hundred thirty patients had non obstructive azoospermia, 53 severe oligozoospermia and 3 necrozoospermia. There were 307 (63%) successful sperm retrieval. Higher testicular volume, lower levels of FSH, and better histological features were predictive for sperm retrieval. The same parameters and younger age were predictive factors for shorter time for sperm recovery. After multivariable analysis, younger age, better semen parameters, better histological features and lower values of FSH remained predictive for shorter time for sperm retrieval while better semen and histology remained predictive factors for successful sperm retrieval. The predictive capacity of a score obtained by summing the points assigned for selected predictors (1 point for Sertoli cell only, 0.33 points for azoospermia, 0.004 points for each FSH mIU/ml) gave an area under the ROC curve of 0.843.

**Conclusions:**

This model can help the practitioner with counseling infertile men by reliably predicting the chance of obtaining spermatozoa with testicular sperm extraction when a repeat attempt is planned.

## Background

The testicular biopsy has been for years one of the crucial investigations in the diagnosis and management of male infertility [1]. The advent of intracytoplasmic sperm injection (ICSI) made possible successful assisted reproduction with sperm derived from the testis in patients with non-obstructive azoospermia (NOA) through techniques of testicular sperm extraction (TESE). One of the major challenge with conventional TESE (cTESE) was to find indicators of the likelihood to recover spermatozoa. From time to time, circulating hormonal markers, testicular ultrasound and testicular biopsy have been used [2]. None of these procedures has proved particularly effective because often, due to the profound heterogeneity of the testicular tissue, spermatozoa were recovered even in the presence of indicators suggestive for adverse outcomes. Nevertheless, biopsy still seemed to be the best predictor of sperm recovery (SR). A further reduction in the use of forecasting techniques occurred with the advent of micro TESE (mTESE). Many groups have argued that the mTESE provides such a high SR compared to other techniques, even in case of severe histological diagnosis like agenesis of the germinal line, that cTESE and as a logical consequence all the procedures to predict the chances of SR, were put aside as techniques of historical interest not relevant to current reproductive technologies [3–7].

However, besides recent studies have questioned the superiority of mTESE compared to cTESE in the resilience of the sperm retrieved [8, 9], the question of being able to provide the patient and the doctor with a system to assess the likelihood of recovering sperm in cases of NOA retains all its meaning, particularly when a reintervention following an unsuccessful sperm retrival attempt is requested. Here we investigated retrospectively the correlations between clinical, laboratory, demographic characteristics, histolgical features and cTESE sperm retrieval outcomes. Predictive values of each one of these characteristics in the SR were scrutinized with the aim of providing elements to make a choice as conscious as possible in case of reintervention.

## Methods

Four hundred eighty-six patients referring to the Centre for Reproductive Medicine, of the European Hospital (Rome, Italy) between january 2005 and june 2016, retrospectively identified to have undergone cTESE and ICSI, were studied. Concurrently with TESE all patients were subjected to testicular biopsy for histological analysis. All patients had a semen sample evaluated in at least two different occasions according to the WHO criteria [10]. Azoospermia was diagnosed when the absence of sperm was observed after 600 g centrifugation and screening at 400× magnification. Cryptozoospermia was one of the conditions considered under the spectrum of NOA [11], defined as the absence of spermatozoa in fresh semen preparations but rare sperm in a centrifuged pellet [10]. Cryptozoospermia occurrence is attributed to fluctuations in spermatogenesis in cases of NOA [11]. Oligozoospermia and necrozoospermia were defined as less than 15 million sperm/ml and less than 5% viable spermatozoa in the ejaculate, respectively. Oligozoospermic patients were submitted to TESE after multiple ICSI failure with poor embryo quality and repeated implantation failure using motile ejaculatory spermatozoa on the basis of the reported better outcome in selected severe oligozoospermic patients when testicular spermatozoa were used [12].

A clinical history was recorded, including history of undescended testis, mumps orchitis, previous genito-urinary infection, radiotherapy, chemotherapy, surgical procedures or exposure to gonadotoxins. A clinical examination included secondary sexual characteristics, testicular size (measured with a Prader’s orchidometer) and consistency, epididymal distension and varicocele. All patients had their serum follicle stimulating hormone (FSH), luteinizing hormone (LH) and testosterone (T) concentrations measured without any hormonal medical therapy within 2 months before cTESE. Karyotype and Y-chromosomal microdeletion analysis were performed on all patients. Patients with AZFa, AZFb, AZFab, AZFbc, AZFabc microdeletions were excluded.

### Surgical technique

The surgical procedure was performed under general anesthesia. After scrotal disinfection, the spermatic cord and the scrotal skin were infiltrated with 8 ml of 7.5 mg/ml ropivacaine hydrochloride (Norepine, ASTA, Milan, Italy). The testicle on which the procedure was started was the one with larger volume. For cTESE, a small (5 mm) equatorial horizontal incision of the albuginea with extrusion of the testicular parenchima and scissors biopsy of approximately 5x2x3mm were performed. If no sperm was found, multiple conventional superficial biopsies (4–8 sampling) on the contralateral testicle was performed following the same procedure. A fragment of the testis removed from the testis used for the cTESE was processed for histology (see below). The surgical procedure was always performed by the same surgeon (GF) and the specimen processed by the same biological team. The time required to find sperm in the tissue was recorded.

### Histology

A fragment of testicular parenchyma (2x2x2 mm) removed from the testis used for cTESE procedure was washed in buffered medium (Quinn’s Advantages Medium with HEPES, SAGE, Cooper Surgical, Pasadena, USA) with 2.5% human serum albumin (HSA, Albutein, Alpha Therapeutic Milan, Italy), fixed in Bouin’s solution (1 ml) and sent to the pathology laboratory. All histological examinations were performed by the same pathologist. Based on the histopathological pattern, testicular histology was classified into: normal spermatogenesis (NormoS); hypospermatogenesis (HypoS, i.e. a reduction in the number of normal spermatogenetic cells); maturation arrest (MA, i.e. an absence of the later stages of spermatogenesis); and Sertoli cell only (SCO, i.e. the absence of germ cells).

### Statistical analysis

Data are presented as mean ± standard deviation or median ± inter quartile range for non-symmetric continuous variables, and as counts and percentages for categorical ones.

The binary outcome (successful SR) was associated with potential predictors through logistic regression, with a Firth bias-reducing correction. The time-to-event outcome (time to successful SR) was associated with potential predictors through (univariate and multivariable) Cox regression modeling. In case of unsuccessfull SR, time-to-event was censored as the time spent on unsuccessful searching. Both for binary and time-to-event outcomes, the final multivariable model was selected by via forward-stagewise selection, based on Akaike Information Criterion (AIC) [13]. All potential predictors were initially considered for inclusion in the multivariable model, but only those leading to significant decrease in AIC (as sequentially considered) were finally included.

The final multivariable linear logistic regression model was used to build a score for successful SR. The points assigned to each indicator or unit for continuous predictors were obtained by rounding regression coefficients. The score obtained was evaluated by means of a receiver operating curve (ROC) analysis for a final model. The area under a curve (AUC) is a measure of predictive power. The value of 0.5 means that predictions are no better than random guessing and the value of 1.0 indicates a (theoretically) perfect test (i.e., 100% sensitive and 100% specific). A *p* < 0.05 was considered as statistically significant and all tests were two-sided. All statistical analyses were performed with the software R version 3.2.0.

## Results

The present study includes 486 patients with a mean age of 37.2 ± 6.5 years (range 20–71 years): 430 with NOA (40 with cryptozoospermia), 53 severe oligozoospermic men who had previously failed to achieve paternity with assisted reproductive technology procedures and 3 with necrozoospermia.

In the NOA group, the mean patient age was 37.2 ± 6.6 years (range 20–71 years); in the severe oligozoospermia group, the mean patient age was 37.3 ± 6.0 (range 27–63); in the necrozoospermia group, the mean patient age was 42.0 ± 2.6 (range 39–44).

Table 1 shows the clinical parameters of the patients stratified according to SR. With the only exception of body mass index [BMI, weight (kg)/height (m^2^)] and circulating T, all the other parameters differed significantly between the patients that experienced successfull SR compared to those that had unsuccessfull SR, including age that was older in the successfull SR group, FSH and LH whose values were higher in the unsuccessfull SR group, and testis volume that was lower in the unsuccessfull SR group.

**Table 1:** Clinical parameters and histopathological features of the patients stratified according to the SR

	Succesfull SR	No SR	*P*
Patients n.	307	179	
Age (years)	38.2±7.05	35.4±4.97	< 0.01
BMI (kg/m^2^)	26.85±6.00	26.51±2.78	NS
FSH (mIU/ml)	15.70±12.22	22.51±12.11	< 0.01
LH (mIU/ml)	6.85±4.86	8.87±5.19	< 0.01
T (ng/ml)	4.57±1.94	4.39±3.11	NS
Testis vol. (right, ml)	9.77±6.46	7.11±4.66	0.010
Testis vol. (left, ml)	9.47±7.41	6.96±3.68	0.021
SCO n. (%)	60 (19.5%)	145 (81%)	< 0.01
MA n. (%)	58 (18.9%)	17 (9.5%)	< 0.01
HypoS n. (%)	132 (43.0%)	17 (9.5%)	< 0.01
NormoS n. (%)	57 (18.6%)	0 (0%)	< 0.01

*SR* sperm retrieval, *BMI* body mass index, *FSH* follicle stimulating hormone, *LH* luteinizing hormone, *T* testosterone, *SCO* Sertoli cell only, *MA* maturation arrest, *HypoS* hypospermatogenesis, *NormoS* normospermatogenesis, *NS* not significant

Table 2 shows the clinical characteristics of the patients stratified according to histology. The sample size for the four groups of histological diagnosis was 205 for SCO, 75 MA, 149 HypoS, and 57 NormoS. Spermatozoa were recovered in 307 out of the 486 patients (63.17%). 60/205 (29.3%) from SCO, 58/75 (77.3%) from MA, 132/149 (88.6%) from HypoS, 57/57 (100%) from NormoS. The 57 NormoS were either oligozoospermic, necrozoospermic or cryptozoospermic. With the exception of BMI and T, all the clinical parameters were significantly different between the groups. Statistical difference in sperm retrieval rate was observed between all the groups with a pattern of increasing likelihood of SR from SCO to MA, HypoS and NormoS. The leves of FSH and LH were progressively lower and testicular volume was higher as much as the histological appearance was improving.

**Table 2:** Clinical characteristics of the patients and SR rate stratified according to testicular histology

	SCO	MA	HypoS	NormoS	*P*
Patients n.	205	75	149	57	
Age (years)	35.64±5.42	37.21±6.67	39.05±7.83	37.57±4.50	< 0.01
Azoospermia n. (%)	196 (95.6%)	60 (80.0%)	121 (81.2%)	13 (22.8%)	< 0.01
Criptozoospermia n. (%)	7 (3.4%)	10 (13.3%)	6 (4.0%)	17 (29.8%)	< 0.01
Oligozoospermia n. (%)	2 (1.0%)	5 (6.7%)	22 (14.8%)	24 (42.1%)	< 0.01
Necrozoospermia n. (%)	0 (0%)	0 (0%)	0 (0%)	3 (5.3%)	< 0.01
BMI (kg/m^2^)	25.86±2.90	27.71±3.54	27.66±7.80	25.5±2.42	NS
FSH (mIU/ml)	24.34±11.65	18.15±11.63	12.66±11.60	11.02±8.81	< 0.01
LH (mIU/ml)	9.80±6.00	7.25±4.08	5.63±3.25	5.18±2.90	< 0.01
T (ng/ml)	4.30±2.85	4.40±2.06	4.75±1.87	4.93±2.26	NS
SR	60 (29.3%)	58 (77.3%)	132 (88.6%)	57 (100%)	< 0.01
Testis vol. (right, ml)	7.24±4.58	7.75±3.39	11.05±8.52	12.38±5.15	< 0.01
Testis vol. (left, ml)	7.17±4.06	8.23±3.69	10.43±10.44	10.56±3.17	NS

*SCO* Sertoli cell only, *MA* maturation arrest, *HypoS* hypospermatogenesis, *NormoS* normospermatogenesis, *BMI* body mass index, *FSH* follicle stimulating hormone, *LH* luteinizing hormone, *T* testosterone, *SR* sperm retrieval, *NS* not significant

The univariate logistic regression analysis showed that four factors were associated with SR including semen analysis, histology, FSH values and testicular volume (Table 3A). As expected, the odds to recover spermatozoa from testicular specimens was significantly higher in both criptozoospermic and oligozoospermic patients compared to azoospermic patients. Analogously, the odds to recover spermatozoa was higher in MA and hypospermatogenic testes compared with testicular tissue specimens affected by SCO. Also significant was the odds of SR for each mIU increase of circulating FSH and for each ml increase of testicular volume. The same variables were significantly associated with the hazard ratio (HR) for time for SR with the exception of oligo-astenozoospermic versus azoospermic (Table 3B).

**Table 3:** Predictive factors of sperm recovery (A) and of time for recovery (B) by cTESE, univariate analysis

A. Sperm recovery			
Predictor variable	OR	95% Cl	*P*
Seminal fluid			
Criptozoospermic vs azoospermic	12.67	4.19–62.31	< 0.01
Oligo-astenozoospermic vs azoospermic	28.80	7.69–256.10	< 0.01
Histology			
MA vs SCO	8.04	4.44–15.19	< 0.01
HypoS vs SCO	18.21	10.41–33.49	< 0.01
FSH	0.96/mIU	0.94–0.97	< 0.01
Testis volume	1.05/ml	1.01–1.11	0.013
B. Time for sperm recovery			
Predictor variable	HR	95% Cl	*P*
Age	1.06/year	1.04–1.08	< 0.01
Seminal fluid			
Criptozoospermic vs azoospermic	3.34	2.05–5.45	< 0.01
Oligozoospermic vs azoospermic	6.64	0.88–46.15	NS
Histology			
MA vs SCO	4.61	2.84–7.49	< 0.01
HypoS vs SCO	9.10	6.03–13.74	< 0.01
FSH	0.95/mIU	0.93–0.96	< 0.01
Testis volume	1.02/ml	1.01–1.04	< 0.01

*OR* odds ratio, *HR* hazard ratio, *FSH* follicle stimulating hormone, *SCO* Sertoli cell only, *MA* maturation arrest, *HypoS* hypospermatogenesis, *NS* not significant

Multiple logistic regression analysis of variables, including semen and histology for SR and age, semen, serum FSH and testicular histology for sperm recovery time revealed that semen and testicular histology were both found to be significant variables to predict successful SR (Table 4A) while age, semen, histology and FSH were significant variables to predict time for sperm recovery (Table 4B).

**Table 4:** Predictive factors of sperm recovery (A) and of time for recovery (B) by cTESE, multivariate analysis

A. Sperm recovery			
Predictor variable	OR	95% Cl	*P*
Seminal fluid			
Criptozoospermic vs azoospermic	8.00	2.24–42.64	< 0.01
Oligozoospermic vs azoospermic	6.55	1.56–63.11	< 0.01
Histology			
MA vs SCO	6.94	3.77–13.30	< 0.01
HypoS vs SCO	16.44	9.31–30.54	< 0.01
B. Time for sperm recovery			
Predictor variable	HR	95% Cl	*P*
Age	1.05/year	1.02–1.07	< 0.01
Seminal fluid			
Criptozoospermic vs azoospermic	1.51	0.87–2.62	NS
Oligozoospermic vs azoospermic	2.05	1.25–3.37	< 0.01
Histology			
MA vs SCO	4.07	2.36–7.02	< 0.01
HypoS vs SCO	6.60	3.96–10.99	< 0.01
FSH	0.98/mIU	0.96–0.99	0.030

*OR* odds ratio, *HR* hazard ratio, *FSH* follicle stimulating hormone, *SCO* Sertoli cell only, *MA* maturation arrest, *HypoS* hypospermatogenesis, *NS* not significant

**Fig. 1:**
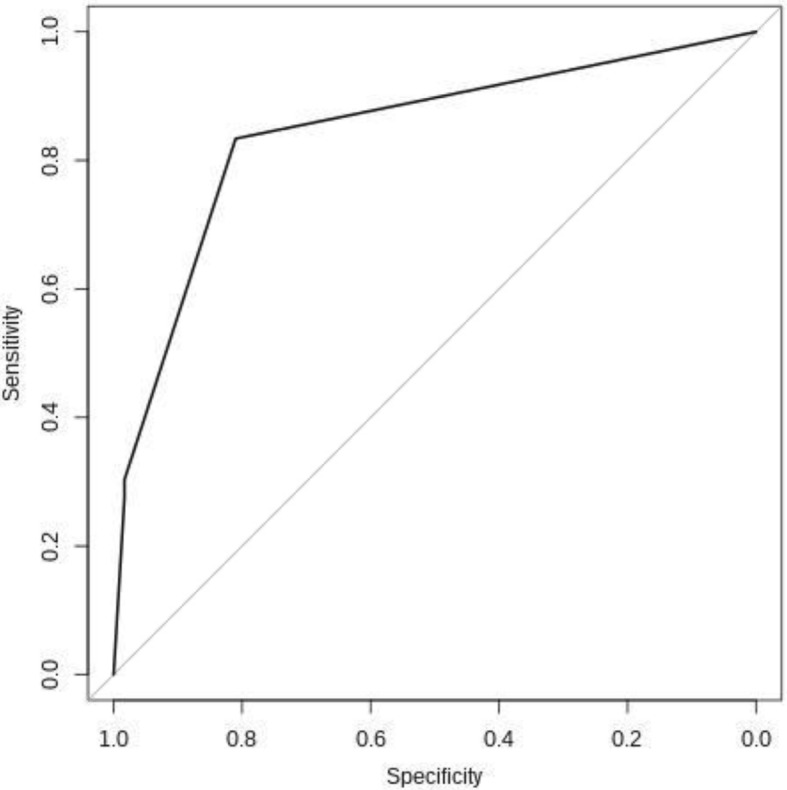
ROC curve of pertinent parameters to discriminate successful and failed cTESE (AUC = 0.843). ROC = Receiver operating characteristic; cTESE = conventional testicular sperm extraction; AUC = area under a curve

We developed a model for the prediction of SR based on a score composed of three variables derived from logistic regression analyses, obtained by summing the points assigned for each predictor (1 point for SCO, 0.33 points for azoospermia and 0.004 points for each FSH mIU). The predictive ability of the score was evaluated by using the area under the ROC curve (Fig. 1) that gave a value of 0.843 with good discriminative performance. Therefore, we identified a cut-off value of the score ≤ 1.24 with a calculated specificity of 83.39% and sensitivity of 81.11% as suggestive of a good chance of SR upon further TESE.

## Discussion

One of the major issues to be addressed in the management of male infertility by TESE is to have an indicator of the likelihood of recovery of sperm from the testis. This information is important for the couple to make a thoughtful choice about whether or not to undertake an in vitro fertilization procedure that can cause physical, psychological and financial consequences. It has recently been called into question the validity of predictive indicators of recovery of sperm from the testes of patients suffering from NOA, in particular the usefulness of diagnostic testicular biopsy [3–7]. The criticisms to the procedure are mainly based on two considerations. The first is that the histological appearance of a bioptic specimen does not mirror the condition of the testicular parenchyma as a whole and therefore it is not worth referring the patient to biopsy because even severe histological features such as SCO, do not rule out the presence of portions of tissue with intact spermatogenesis, generating false negatives. The second is that the mTESE, according to the proponents of the technique, substantially lowers the risk of not detecting preserved spermatogenesis areas, if present, ensuring almost total certainty of potential sperm recovery, reducing significantly the usefulness of diagnostic biopsy. These arguments have, in turn, limitations. It is clear that whatever the procedure, either diagnostic or therapeutic, it will never provide the absolute certainty of the absence of germ cells for fertilization, because in no case the entire tissue can be analized and the risk not to identify areas of tissue with intact spermatogenesis can not be eliminated. It is not clear, moreover, what is the real advantage of the mTESE in terms of recovery compared with cTESE because recent studies have not confirmed its superiority in all circumstances [8, 9, 14, 15]. Although, pseudo-randomized prospective data show more favourable sperm retrieval in NOA for mTESE compared to cTESE, especially in histological patterns of patchy spermatogenesis such as SCO, in patients with uniform histological patterns such as MA, the outcome of mTESE seems less favourable [14]. No secure clinical predictors of sperm retrieval are demonstrated for both procedures and clinical complication rate seems not to differ according to a systematic review [14]. A further systematic review with meta-analysis took into consideration fifteen studies with a total of 1890 patients [15]. In a direct comparison, performance of mTESE was 1.5 times more likely (95% confidence interval 1.4–1.6) to result in successful SR as compared with cTESE. The essential weakness of the studies and meta-analysis on mTESE is the lack of proper control groups. Also the studies comparing the success rate of mTESE against cTESE should be viewed with caution because due to the extreme variability of the spermatogenic process, clearly visible also in patients with normal spermatogenesis (seminal parameters in normozoospermic patient may vary profoundly a few days away), the success or failure of SR can change significantly from day to day.

There are only few exceptions to this methodological limit, namely studies in which both mTESE and cTESE were performed during the same recovery procedure in the same patient in comparison studies. Franco et al. showed that the outcome of mTESE did not improve SR as compared to single cTESE biopsy on the same testicle or to multiple contralateral cTESE [8]. Analogously, Marconi and colleagues found no difference between mTESE and cTESE in terms of sperm retrieval rates [9].

Whatever the best technique to recover spermatozoa, the opportunity to offer to both doctors and patients a likely indicator of success of SR rate is important. A situation that can occur in case of reoperation following a failure of TESE, provided that the information coming from the microscopic analysis of a testicular fragment is available.

The evidence for success rates of repeat sperm retrieval surgery in men with NOA is based on a very small number of retrospective case series with varying patient selection criteria and methodologies. The success rate of repeat TESE varied from 30% [16] to 41.6% in the first repeat attempt and the success rate increased to 100% for two patients with six attempts [17], there are limitations of this evidence as only 2 out of 628 patients in the study reached six attempts, hence it is difficult to generalise.

One retrospective case series of repeat mTESE showed a success rate of 82% [18]. The study identified lower FSH level and larger testicular volume to have a predictive value in determining the success of a second attempt. The findings of the study are limited by its retrospective, nonrandomized, non-controlled nature. In summary, there is low level evidence from retrospective case series that the cumulative success rate of repeat sperm retrieval increases with increasing numbers of attempts and is higher in males who have had a previous successful attempt. The results are not substantiated by other studies, hence the replicability of these results in other patients or settings is limited. These considerations substantiate the utility of the testicular biopsy, togheter with other potential predictors of SR, in dealing with assisted reproductive techniques that require TESE when further attempts of TESE are planned. Accordingly, our data suggest not to miss the opportunity to collect a testicular fragment in the course of TESE to perform histologic analysis. Clearly, histology should not be included as a predictive factor of SR at first TESE attempt since the TESE specimen, either conventional or micro, not a prior pure diagnostic biopsy is what is read for histology and a patient will know of succesful sperm retrieval before histology results are even available. Nevertheless, the diagnostic biopsy during cTESE or even mTESE, along with other indicators may help to make an informed choice in the hypothesis of a subsequent new attempt of SR. Therefore, the allegation that it is time to put testis biopsy aside as a technique of historical interest not relevant to current reproductive technologies should be called into question and the opportunity to collect a testicular specimen for histology during the course of TESE, for a better stratification of the SR chance in case of a subsequent attempt following a failure, should be taken. Accordingly, recent published evidences support the reliabilty of diagnostic biopsy as a predictor of positive sperm retrieval in men with NOA [19–23].

By performing TESE, the laboratory personnel should allow themselves enough time to dissect the testicular tissue and retrieve the sperm, particularly in the case of NOA patients, for whom spermatogenesis may be severely impacted and in whom it may take a long time to find sperm in the tissue [24]. Interestingly, the data presented here provide an estimate of what factors are expected to make sperm retrieval faster or slower. As expected, age, worst semen and histology and higher FSH values were all negative predictive factors in the multivariate analysis.

Our study does have limitations. Most importantly, a control population of men who were submitted to mTESE was not available. Other limitation includes the retrospective nature of the study.

## Conclusions

Our data enable to construct a score which helps to provide a good approximation of the probability of SR with cTESE in case of reoperation because of failure in recovery of sperm, provided that a biopsy for microscopic analysis is harvested during the first TESE attempt. The biopsy, along with other parameters, enables a customization of the prognosis that would otherwise rely solely on literature data that often extend over a very wide range and still have the limitation of being strongly influenced by skill or experience of the team that created them and does not apply to all the peculiar situations that can bump into real life. Providing patients with a personalized, more clinically meaningful estimate of their likelihood of SR can aid in counseling and decrease anxiety for the patient and treating physician. Furthermore, consideration should be given to the variables found to be involved into the time required for sperm recovery. This individualized estimate is likely to improve the complex organisation of assisted reproductive technology procedures that may require multiple attempts, including repeated TESE.

## Abbreviations

AIC: Akaike Information Criterion
AUC: Area under a curve
BMI: Body mass index
cTESE: Conventional TESE
FSH: Follicle stimulating hormone
HR: Hazard ratio
HypoS: Hypospermatogenesis
LH: Luteinizing hormone
MA: Maturation arrest
mTESE: Micro TESE
NOA: Non-obstructive azoospermia
NormoS: Normal spermatogenesis
ROC: Receiver operating curve
SCO: Sertoli cell only
SR: Sperm recovery
T: Testosterone
TESE: Testicular sperm extraction
